# Aerobic Barley Mg-protoporphyrin IX Monomethyl Ester Cyclase is Powered by Electrons from Ferredoxin

**DOI:** 10.3390/plants9091157

**Published:** 2020-09-08

**Authors:** David Stuart, Malin Sandström, Helmy M. Youssef, Shakhira Zakhrabekova, Poul Erik Jensen, David W. Bollivar, Mats Hansson

**Affiliations:** 1 Department of Biology, Lund University, Sölvegatan 35B, 22362 Lund, Sweden; david.stuart@biol.lu.se (D.S.); malinsandstrom8@hotmail.com (M.S.); helmy.youssef@biol.lu.se (H.M.Y.); Shakhira.zakhrabekova@biol.lu.se (S.Z.); 2 Faculty of Agriculture, Cairo University, Giza 12613, Egypt; 3 Department of Food Science, University of Copenhagen, Rolighedsvej 26, DK-1958 Frederiksberg, Denmark; peje@food.ku.dk; 4 Department of Biology, Illinois Wesleyan University, Bloomington, IL P.O. Box 2900, USA; dbolliva@iwu.edu

**Keywords:** *acsF*, *bchE*, *CHL27*, chlorophyll biosynthesis, *CRD1*, FNR, *Hordeum vulgare*, XanL, *Xantha-l*

## Abstract

Chlorophyll is the light-harvesting molecule central to the process of photosynthesis. Chlorophyll is synthesized through 15 enzymatic steps. Most of the reactions have been characterized using recombinant proteins. One exception is the formation of the isocyclic E-ring characteristic of chlorophylls. This reaction is catalyzed by the Mg-protoporphyrin IX monomethyl ester cyclase encoded by *Xantha-l* in barley (*Hordeum vulgare* L.). The *Xantha-l* gene product (XanL) is a membrane-bound diiron monooxygenase, which requires additional soluble and membrane-bound components for its activity. XanL has so far been impossible to produce as an active recombinant protein for in vitro assays, which is required for deeper biochemical and structural analyses. In the present work, we performed cyclase assays with soluble and membrane-bound fractions of barley etioplasts. Addition of antibodies raised against ferredoxin or ferredoxin-NADPH oxidoreductase (FNR) inhibited assays, strongly suggesting that reducing electrons for the cyclase reaction involves ferredoxin and FNR. We further developed a completely recombinant cyclase assay. Expression of active XanL required co-expression with an additional protein, Ycf54. In vitro cyclase activity was obtained with recombinant XanL in combination with ferredoxin and FNR. Our experiment demonstrates that the cyclase is a ferredoxin-dependent enzyme. Ferredoxin is part of the photosynthetic electron-transport chain, which suggests that the cyclase reaction might be connected to photosynthesis under light conditions.

## 1. Introduction

Chlorophylls are the most abundant light-harvesting pigments on Earth and essential for the process of photosynthesis. They belong to a large family of tetrapyrrole molecules also including hemes, bilins, and corrins. Chlorophylls are distinguished from the other tetrapyrroles by a centrally chelated magnesium ion and a fifth isocyclic ring, named the E ring. Chlorophyll biosynthesis is a major anabolic pathway divided into 15 enzymatic steps, each catalyzed by a unique enzyme [1]. The initial nine biosynthetic reactions are common to both chlorophyll and heme. In the first unique step of the chlorophyll biosynthetic pathway, Mg^2+^ is inserted into protoporphyrin IX. Subsequently, a methyl group is transferred to the carboxyl group of the propionate on the C ring of Mg-protoporphyrin IX, generating Mg-protoporphyrin IX monomethyl ester (MPE), which is the substrate of the MPE cyclase in focus of the present study. The cyclase catalyzes the formation of the isocyclic E ring by insertion of oxygen and attaching the methylated propionate to the methene bridge between pyrrole rings C and D, forming protochlorophyllide. Chlorophyll is obtained after additional reactions involving a light-dependent oxidation of protochlorophyllide to chlorophyllide, reduction of the vinyl group on the B ring, and, finally, addition of a polyisoprene tail [1].

Most steps for chlorophyll biosynthesis have been studied using recombinant proteins in defined reactions, but the MPE cyclase is an exception. Instead, present knowledge concerning the cyclase is based on studies using fractionated cell extracts and genetic analyses of bacteria, algae, and plants [2,3,4,5,6,7,8,9,10,11]. Two distinct enzymes have been identified that catalyze the cyclase reaction, originally distinguished by the source of the incorporated oxygen. The enzyme that catalyzes the cyclase reaction in the absence of molecular oxygen (anaerobic) derives the oxygen from water and is encoded by *bchE* in facultative photosynthetic bacteria like *Rhodobacter sphaeroides* [12]. A reliable in vitro assay for this enzyme has not been developed yet. In the oxygen-requiring (aerobic) reaction, one of the oxygen atoms from molecular oxygen is incorporated into the substrate and the other is reduced to water [10,12]. Thus, the anaerobic enzyme functions as a hydratase, whereas the aerobic cyclase is an oxygenase. The aerobic cyclase was first identified in the purple nonsulfur photosynthetic bacterium *Rubrivivax gelatinosus* and named AcsF [6]. *R. gelatinosus* also has the *bchE* gene to enable photosynthesis under various oxygen conditions [13]. The discovery of *acsF* opened the possibility to identify the orthologous gene in other organisms, e.g., *Hordeum vulgare* and *Cth1* in *Chlamydomonas reinhardtii* [14,15]; *chlA1*, *chlA2*, *cycI*, and *cycII* in *Synechocystis* sp. PCC 6803 [16,17]; *CHL27* in *Arabidopsis thaliana* [9]; and *Xantha-l* in barley (*Hordeum vulgare* L.) [7,18].

The aerobic cyclase belongs to the family of diiron carboxylate-bridged proteins characterized by the iron-binding motif E-X_n_-E-X-X-H-X_n_-E-X_n_-E-X-X-H [19]. Detailed studies on the enzyme have been impaired by the absence of a recombinant expression system. Fractionation of cell extracts of cucumber (*Cucumis sativus* L.) [20,21], *C. reinhardtii*, *Synechocystis* [22], and barley [7,23] revealed that cyclase activity requires both additional soluble and membrane-bound fractions, but all involved components have not been identified. The discovery of Ycf54 and LCAA in *Synechocystis* [24] and tobacco *Nicotiana tabacum* L. [2], respectively, indicated that this protein affected the cyclase enzyme function in organisms that perform oxygenic photosynthesis, although the protein is not required for cyclase activity by the *acsF*-encoded aerobic cyclase found in organisms that perform anoxygenic photosynthesis [3]. Despite the lack of an active recombinant enzyme in vitro, a reaction mechanism of the aerobic cyclase has been proposed. These models suggested transformation via a β-oxidation of the propionate side-chain, similar to the β-oxidation of fatty acids, via β-hydroxy and β-keto intermediates [11,25,26]. Thus, it can be speculated that the additional soluble and membrane-associated components are involved in the redox chemistry of the reaction.

In the present work, we report a completely recombinant assay based on the barley *Xantha-l* gene product encoding the aerobic cyclase XanL. Production of active XanL strictly requires co-expression of *Ycf54*. We further demonstrate that assays performed with barley extract are inhibited by antibodies raised against Fd and ferredoxin-NADPH oxidoreductase (FNR). Finally, cyclase activity was obtained by combining the recombinantly expressed XanL with Fd and FNR. Our work establishes all components necessary for the functional recombinant cyclase and serves as a platform for further detailed studies on the aerobic enzyme, where Fd is the direct electron donor.

## 2. Results

### 2.1. Inhibition of Cyclase Activity with Antibodies

**Figure 1:**
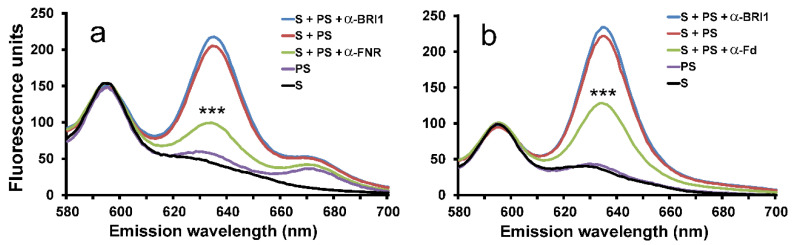
Effect of antibodies on enzymatic cyclase activity. Soluble (S) and solubilized membrane (PS) fractions of barley plastids were combined to obtain cyclase activity (red). Addition of antibodies raised against ferredoxin-NADPH oxidoreductase (FNR) (**a**, green) or Fd (**b**, green) significantly inhibited activity. Assays with addition of antibodies raised against barley brassinosteroid receptor BRI1 were used as controls to ensure that there was no general inhibitory effect of antibodies. Three parallel assays were performed, and the displayed curves are averages of the three replicates. ***, *p* < 0.001.

Fd and FNR are plastid-localized redox components, and we sought to test their role in the cyclase reaction. Antibodies are known to inhibit enzymatic reactions by steric interference [27], and antibodies against FNR have previously been shown to be effective FNR inhibitors [28]. Therefore, we used antibodies against either FNR (α-FNR) or Fd (α-Fd) to test for inhibition of cyclase activity in assays using well-established barley plastid fractions prepared according to Bollivar et al. [23]. Addition of α-FNR or α-Fd resulted in a severe reduction in enzymatic activity (Figure 1). To ensure that there was no general inhibitory effect of antibodies, a brassinosteroid receptor (BRI1) antibody directed toward the barley brassinosteroid receptor [29] was included as a control but had no effect on cyclase activity (Figure 1).

**Figure 2:**
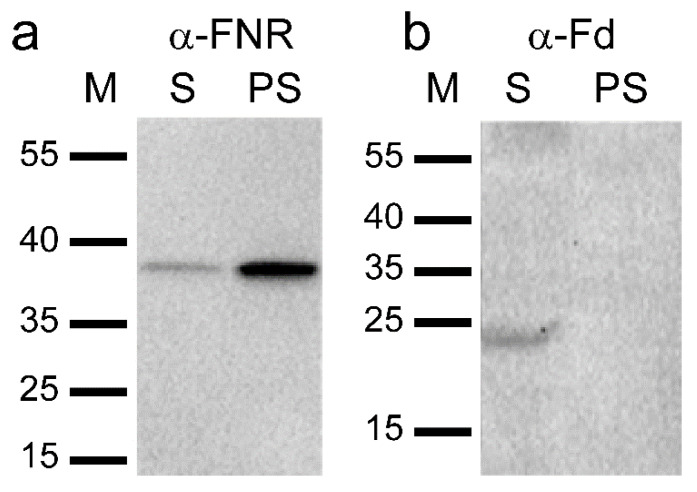
Immunoblot analysis of the soluble (S) and solubilized membrane (PS) fractions used in the assays. (**a**) FNR is predominantly detected in PS. (**b**) Fd is only detected in S.

**Figure 3:**
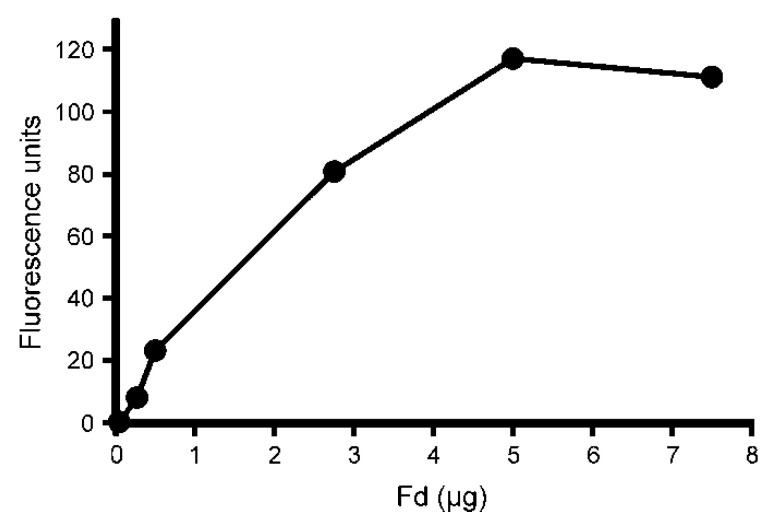
The effect of spinach ferredoxin (Fd) on cyclase activity. Assays were performed with an increasing amount of Fd added and a constant amount of solubilized membrane (PS) fractions of barley plastids. Fd could replace the soluble plastid fraction (S). Product formation increased with the amount of added Fd until saturation at 5 µg added Fd (y = 23.3x + 6.35, R^2^ = 0.980, *p* = 0.0012). 1 μg Fd corresponds to 97 pmole.

As the cyclase enzyme system from barley plastids was inhibited by the FNR and Fd antibodies, thus suggesting a role for these proteins in the cyclase reaction directly, immunoblot analysis using the same antibodies was performed on the soluble and membrane fractions used for the cyclase assays. FNR was primarily found in the membrane fraction though a small amount was also detected in the soluble fraction (Figure 2a). Fd, on the other hand, was only detected in the soluble fraction and was, therefore, a likely candidate for the previously unidentified soluble component (Figure 2b). Therefore, commercially available spinach (*Spinacia oleracea*) Fd was tested in cyclase assays to see if it could replace the soluble barley plastid fraction. When spinach Fd was combined with the membrane fraction of barley plastids, activity was indeed observed. The observed cyclase activity increased with Fd concentration (Figure 3). The results clearly demonstrate that Fd is the necessary soluble component of the cyclase enzyme system.

### 2.2. Development of a Recombinant Cyclase Assay

**Figure 4:**
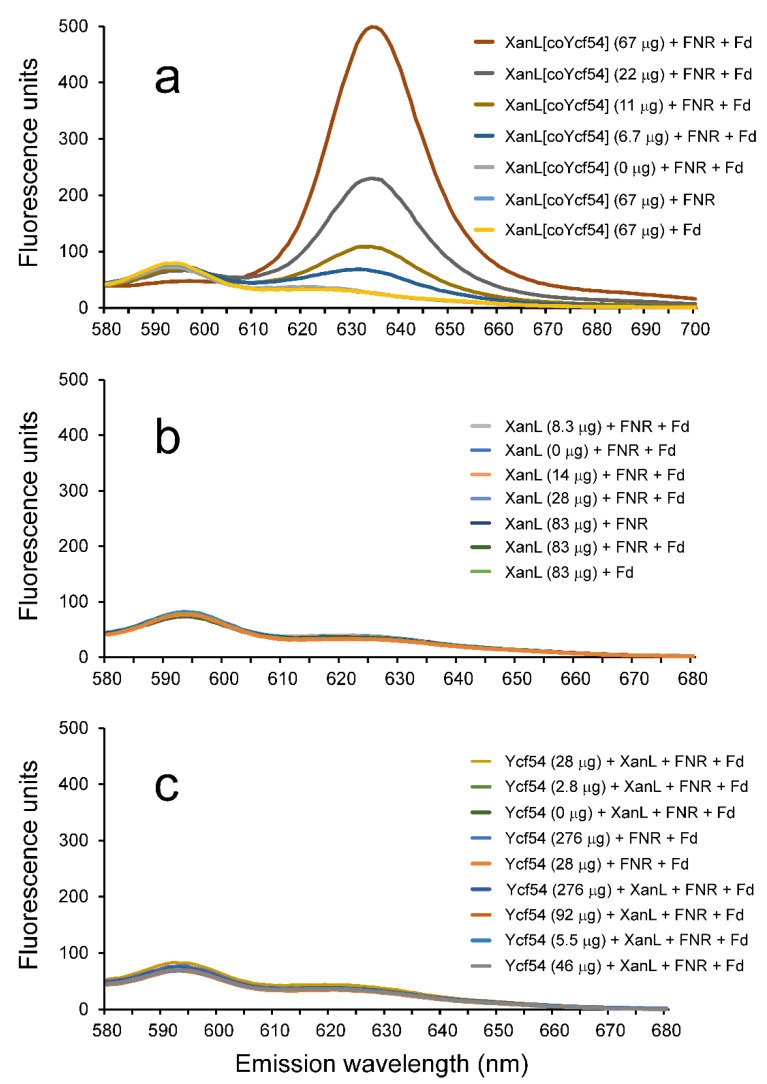
Enzymatic activity of recombinant XanL in combination with spinach Fd (ferredoxin) and spinach FNR (ferredoxin-NADPH oxidoreductase). Used concentrations of Fd and FNR were 0.5 µg/μL and 0.75 milli-units/μL, respectively. (**a**) Cyclase activity assays with recombinant XanL co-expressed with Ycf54 (XanL[coYcf54]). Product formation increased linearly with the amount of added XanL[coYcf54] (y = 6.99x + 12.7, R^2^ = 0.983, *p* = 0.0077). No activity was obtained when Fd or FNR was omitted from the assay. (**b**) Assays performed with recombinant XanL expressed without Ycf54. No activity was detected. (**c**) Assays performed with recombinant XanL (83 μg) and Ycf54 expressed separately. No activity was detected.

In a recent study, heterologous expression of *R. gelatinosus acsF* in *Escherichia coli* demonstrated that cyclase activity could occur in vivo in this non-photosynthetic organism [3]. While this study suggested that any remaining parts of the enzyme are commonly present in *E. coli* cells, it did not fully define the essential components necessary for a completely recombinant system where all required partners are present. Based on the evidence presented above, it seemed that all that was missing for a reconstituted enzyme assay with defined components was an electron transfer system composed of Fd and FNR. However, attempts to simply produce the recombinant XanL protein and recombinant Ycf54 protein, separately, and then combine them with Fd and FNR in an in vitro assay did not result in any activity (Figure 4c). Given that Ycf54 did not show similarity to any previously described enzymes, it seemed plausible that the role of this protein was not catalytic but rather structural. Therefore, expression constructs using a vector that allows for co-expression of two genes were created. In the first plasmid construct, *Xantha-l* was placed in cloning site one (producing a His-tagged protein, XanL[coYcf54]) and *Ycf54* in cloning site two (without a tag). As controls, a second plasmid construct was created that had only *Xantha-l* present in cloning site one (producing His-tagged XanL). A construct for expression of *Ycf54* alone was already available [23]. 

**Figure 5:**
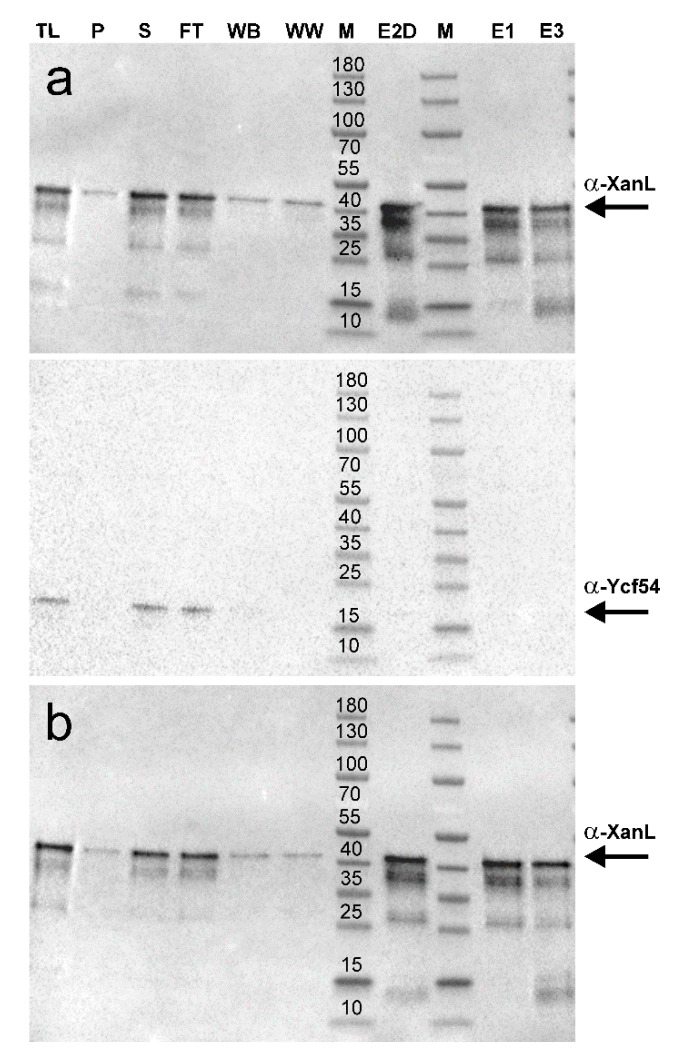
Immunoblots of recombinant barley XanL and Ycf54 produced in *E. coli* ArticExpress (DE3). (**a**) His-tagged XanL and non-His-tagged Ycf54 were co-produced from plasmid pETDuet-1. (**b**) Only His-tagged XanL was produced from pETDuet-1. TL, total lysate; P, pellet; S, supernatant; FT, flow through; WB, wash with binding buffer; WW, wash with wash buffer; M, marker; E2D, elution fraction 2 (desalted); M, marker; E1, elution fraction 1; E3, elution fraction 3. The molecular weight of the marker proteins is indicated (kDa). To ensure comparable loading between experiments, samples S, E2D, E1, and E3 were standardized to a protein concentration of 1 mg/mL prior to loading. Samples TL, P, and FT were loaded on an equal volume basis as the corresponding S.

Enzymatic assays combining spinach Fd and FNR with XanL[coYcf54] showed high activity (Figure 4a), whereas no enzymatic activity was detected in assays with recombinant XanL that was produced alone without Ycf54 (Figure 4b). Immunoblot analyses were performed and showed a similar amount of XanL produced from both expression constructs (Figure 5). As it was possible that Ycf54 was co-purifying with XanL, immunoblot analyses to detect Ycf54 levels were performed. Only trace amounts, if any, of Ycf54 were co-purified along with XanL when they were co-expressed (Figure 5a). Assays were also performed with recombinant XanL and Ycf54 that had been produced separately and then combined in the assay; however, no enzymatic activity could be detected (Figure 4c). Overall, this suggests that Ycf54 is not required for catalysis but fulfills a role during production of XanL within the *E. coli* cell. This conclusion is also supported by a very recent study of *Synechocystis*, *Chlamydomonas*, and Arabidopsis cyclases showing that the in vivo cyclase activity in *E. coli* was dependent on the co-expression of Ycf54 [30]. In our control experiments, it was further shown that activity was only seen when XanL[coYcf54] was combined with both Fd and FNR, and no activity was detected when either Fd or FNR was omitted from the reaction, nor assays with Fd and FNR but without XanL[coYcf54] reinforcing the direct role of both Fd and FNR in the catalytic cycle.

## 3. Discussion

**Figure 6:**
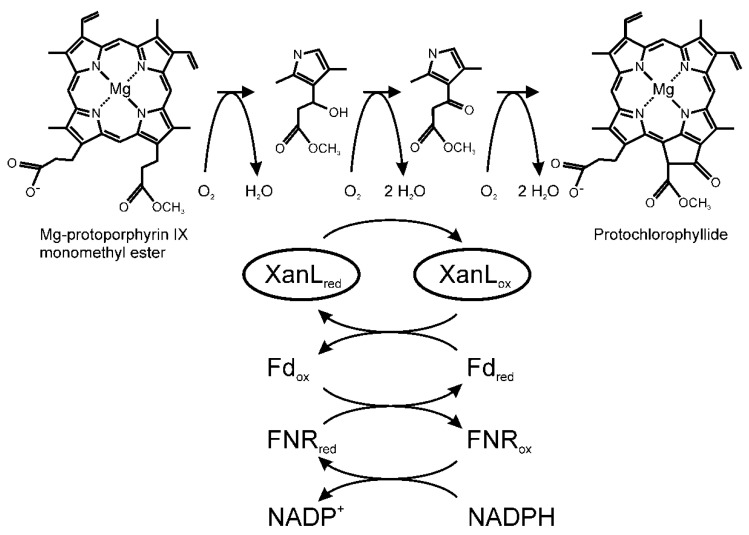
Reaction mechanism of the aerobic cyclase reaction. The cyclase reaction is a six-electron redox reaction suggested to proceed via β-hydroxy and β-keto intermediates. We showed that the electrons are provided by Fd. In the present study, we used NADPH as the source of electrons transferred to Fd via FNR. In green tissue, Fd can also be reduced by photosystem I.

In the mid-1980s, Wong et al. were the first to report that both soluble and membrane-associated chloroplast components are required for the cyclase enzyme [11]. The cyclase is a carboxylate-bridged diiron monooxygenase [31] and, as such, it requires molecular oxygen as well as a reductant to supply electrons [10,31]. However, due to previous difficulties in producing active recombinant cyclase, the enzyme is poorly understood and its electron donor has so far not been identified. In oxygenic photosynthesis, Fd transfers electrons to FNR for production of NADPH. The reverse reaction can also occur [32]. Recently, it was proposed that FNR could act as a direct electron donor to the cyclase or create an NADPH-rich environment at the cyclase complex [33]. Our in vitro assay requires Fd, FNR, and NADPH for activity, which suggests that Fd is the true donor of electrons to XanL as cyclase activity was not obtained with only NADPH added or with NADPH in combination with FNR. By identifying Fd as the unknown soluble component and showing that the cyclase is an Fd-dependent enzyme, we have answered one of the key questions about basic plastid physiology that has been an enigma for almost 40 years, and we can expand the current model of the aerobic cyclase reaction with the electron donor components (Figure 6).

The early biochemical experiments suggested a reaction mechanism via a β-oxidation of the propionate side-chain, similar to the β-oxidation of fatty acids [11,25,26]. It was shown that both the β-hydroxy and β-keto intermediates could function as substrates for the aerobic cyclase [11,34]. While the multi-step nature of the reaction makes investigating individual steps challenging, previous studies have shown that at least the first and last steps require both molecular oxygen and a reductant [10,11,21,34]. The second step, oxidation of the hydroxyl group to a keto group, is likely to have the same requirements. One possible scenario for this middle reaction is that the hydroxylation reaction is simply performed again to produce a geminal diol intermediate that spontaneously dehydrates to form the β-keto intermediate in a fashion similar to that which has been proposed for the chlorophyllide *a* oxygenase reaction [35], although this mechanism has yet to be verified for either enzyme. What has been shown, however, is that only one of the stereoisomers of the β-hydroxy intermediate is an active substrate for the cyclase, which indicates that the first hydroxylation must be stereospecific [34]. Which of the two isomers is the true intermediate has yet to be determined.

The evidence from this study and those supported by other recent studies [3,4,30,36] indicate that the accessory protein Ycf54 is unlikely to be directly involved in the catalytic reaction but likely facilitates proper maturation of the cyclase. One possibility is that Ycf54 facilitates proper folding and maturation of the XanL protein. This hypothesis is in agreement with a recent study showing that a *ycf54* knockout in the cyanobacterium *Synechocystis* PCC 6803 accumulates a low level of chlorophyll and has reduced levels of the XanL orthologue [36]. It was further shown that the formation of protochlorophyllide in vivo in *E. coli* cells expressing the genes of magnesium chelatase, Mg-protoporphyrin IX methyl transferase, and the XanL orthologues of *Synechocystis*, *Chlamydomonas*, and Arabidopsis was strictly dependent on the co-expression of Ycf54 [30]. Presently, only limited amounts of active XanL can be produced facilitated by the co-expression of Ycf54, as shown in our present study and also reported by Chen and Hunter [30]. Thus, further refinement of the recombinant expression systems is required, which is likely to also deepen our understanding of the role and mechanism of Ycf54 in the process of XanL maturation. Accessory proteins connected to various binuclear iron monooxygenases have been reported. For example, a propane monooxygenase encoded by *mimABCD* in *Mycobacterium goodie* required co-expression of *mimG* to be obtained in active form [37]. The *mimG* gene product is similar to the chaperonin GroEL. Another gene, *phk*, encoding an accessory protein of a phenol hydroxylase of *Pseudomonas* sp. OX1, did not appear to be required for the production of active recombinant phenol hydroxylase but, rather, involved in increasing the affinity of the hydroxylase for iron [38]. It is obvious that accessory proteins are common in binuclear iron monooxygenases, and they might shed light on the function of Ycf54 in the process of XanL maturation, although they show no sequence similarity.

While the reductant in our assay system is NADPH, the source of reducing electrons may well be different in planta and even at different developmental stages. In green tissue, Fd is mainly reduced by photosystem I as a result of linear electron transport. Thus, under light conditions, Fd might connect photosynthesis with chlorophyll biosynthesis at the cyclase reaction step. A barley mutant, *viridis-zb.63*, which has only 2% photosystem I activity compared to wild type, behaves like a cyclase mutant, i.e., accumulates the cyclase substrate MPE when fed the chlorophyll biosynthetic precursor 5-aminolevulinic acid [8,39]. In this context, greater amounts of MPE are accumulated by plants incubated in the light as opposed to darkness [8]. This result can be explained if the cyclase receives electrons from photo-reduced Fd in the light, whereas in darkness, Fd would be reduced by FNR. Another stage where Fd is likely to be reduced by FNR is during de-etiolation of seedlings as there are not yet any active photosynthetic complexes that could photoreduce Fd. In both cases, Fd is reduced by FNR with electrons from NADPH generated in the pentose phosphate pathway. Identification of the required components and establishment of an in vitro assay using recombinant XanL have set the stage for future studies to decipher the structure and biochemistry of this enigmatic enzyme, as well as the source of electrons during different developmental stages and light conditions.

## 4. Materials and Methods

### 4.1. Barley Plastid Preparation

Barley (*Hordeum vulgare* L.) etioplasts were prepared and fractionated into soluble and solubilized membrane fractions from cultivar Bonus as described previously [23].

### 4.2. Cyclase Assays

The magnesium protoporphyrin IX monomethyl ester (MPE) substrate for cyclase assays was prepared by the method of [40]. Enzymatic activity assays were performed as described in Bollivar et al. [23] with minor alterations. Unless otherwise specified, the reaction conditions for assays containing barley plastid fractions were 20 mM Tricine and 10 mM HEPES pH 8.1, 1 mM EDTA, 25 mM MgCl_2_, 1 mM DTT, 10 mM glucose-6-phosphate, 0.03 units/µL glucose-6-phosphate dehydrogenase, 0.5 mM NADPH, 90 µg/µL catalase, 0.026% Triton X-100, and 10 µM MPE. Reactions with recombinant XanL were the same except that the EDTA was omitted and contained 0.75 milli-units/μL *Spinacia oleracea* FNR (Sigma-Aldrich, Stockholm, Sweden) and 0.5 µg/μL *S. oleracea* ferredoxin (Sigma-Aldrich, Stockholm, Sweden). Assays were incubated in the dark at 30 °C and 750 rpm for one hour in a Thermomixer comfort (Eppendorf Nordic A/S, Hørsholm, Denmark). Assays were stopped by addition of 80% acetone with 0.32% NH_3_ and centrifuged for 5 min at 29,000× *g* to pellet-precipitated proteins. Formation of protochlorophyllide was measured using an RF-5301 PC spectrofluorophotometer (Shimadzu, Kista, Sweden ) with an excitation wavelength of 440 nm and an emission spectrum between 570 and 700 nm with slit widths of 10 nm for both excitation and emission. Product formation was estimated as the relative fluorescence emission at 634 nm for samples minus the emission for the negative control sample. The linear model, lm(), function in R version 3.6.1 was used for statistical comparisons between categorical treatments, as well as for fitting regression lines to numerical treatments.

### 4.3. Antibodies Used

Polyclonal rabbit antibodies were raised against barley proteins XanL [23], Fd [41], FNR [42], and BRI1 [29]. The polyclonal rabbit antibodies against Ycf54 were produced by immunization with the synthetic peptide CAETVEEALASNPAEL linked to KLH (Keyhole limpet hemocyanin) as a carrier protein (Agrisera, Vännäs, Sweden).

### 4.4. Plasmid Constructs

The plasmids pETDuet-1HvXanL and pETDuet-1HvXanLYcf54 containing *Xantha-l* and *Ycf54* that were codon-optimized for expression in *E. coli* were from GenScript Biotech Corporation (Leiden, The Netherlands). The *Xantha-l* gene was placed in multiple cloning site one between the *Hind*III and *Not*I sites, while the *Ycf54* gene was placed between the *Nde*I and *Bgl*II sites. In order to improve purification, the initial constructs were modified by PCR-based mutagenesis [43], resulting in a His tag with 10 residues instead of 6, as well as insertion of a TEV site between the tag and the *Xantha-l* gene. Constructs were verified by Sanger sequencing after mutagenesis. The primers used for mutagenesis can be found in Table 1. For expression and purification of Ycf54, the plasmid pET15bYcf54 was used [23].

### 4.5. Protein Production

Plasmids were transformed into *E. coli* ArcticExpress (DE3). Overnight cultures were grown at 37 °C at 200 rpm in LB media supplemented with 100 µg/mL ampicillin, 20 µg/mL gentamycin, and 1% glucose. The next day, 6 × 1 L LB in 5 L baffled flasks supplemented with antibiotics were inoculated to an OD_600_ of 0.1. The flasks were placed in a Multitron Standard incubator (Infors HT, Farsta, Sweden) at 37 °C at 200 rpm for 20 min after which the temperature was changed to 15 °C and the cultures were allowed to grow as the temperature gradually decreased. After approximately 3 h when the cultures had an OD_600_ between 0.5 and 0.6, the cultures were induced with IPTG to a final concentration of 1 mM. Cultures expressing *Xantha-l* were also supplemented with solid FeSO_4_ to a final concentration of 1 mM at the time of induction. Cultures were then grown for an additional 68 h before the *E. coli* cells were harvested by centrifugation. Cell pellets were then frozen at −20 °C until use.

### 4.6. Protein Purification

For purification of XanL, the cell pellet from 2 L culture was re-suspended to 35 mL in binding buffer (20 mM imidazole, 500 mM NaCl, 20 mM Tris-HCl pH 8.0, and 15% (*v*/*v*) glycerol) with a few crystals of lysozyme and DNase I added. *E. coli* cells were lysed by passage through a French pressure cell three times at 12.4 MPa followed by centrifugation at 48,384× *g* for 30 min at 4 °C. The supernatant was then loaded on a 1 mL HisTrapFF crude column (GE Healthcare, Helsingborg, Sweden) equilibrated with binding buffer. The column was then washed with 25 mL binding buffer followed by 25 mL wash buffer (45 mM imidazole, 500 mM NaCl, 20 mM Tris-HCl pH 8, and 15% (*v*/*v*) glycerol). The proteins were eluted with elution buffer (250 mM imidazole, 500 mM NaCl, 20 mM Tris-HCl pH 8, and 15% (*v*/*v*) glycerol) and collected in 1 mL fractions. The elution fraction with the highest protein concentration was desalted over a NAP-10 column into 50 mM Tris-HCl pH 8.0 with 15% (*v*/*v*) glycerol. The protein was then stored at −80 °C until use. Protein concentration was estimated using Bradford reagent (BioRad, Solna, Sweden) using bovine serum albumin fraction V as a standard. Purification of Ycf54 using cell pellets from 1 L culture followed the same procedure except that glycerol was not present in the buffers during purification and the buffers were supplemented with 1 mM DTT. In addition, the column was only washed with 15 mL binding buffer and 10 mL wash buffer. The purified Ycf54 was desalted as above into 50 mM Tris-HCl pH 8.0, 15% (*v*/*v*) glycerol, and 1 mM DTT. Ycf54 was not stable when frozen and was used the same day it was purified. All protein purifications were performed at 4 °C.

### 4.7. SDS-PAGE and Immunoblotting

Proteins were separated on 4–15% Mini-Protean TGX precast gels (BioRad). Molecular weight markers (PageRuler^TM^ Prestained Protein Ladder) were from Thermo Scientific. Gels were stained with Bio-Safe Coomassie G-250 (BioRad, Solna, Sweden ) or, when used for immunoblots, transferred onto 2 µm PVDF membranes (BioRad). Membranes were blocked for 1 h in 5% non-fat powdered milk in PBS (137 mM NaCl, 2.7 mM KCl, 10 mM Na_2_HPO_4_, 1.8 mM KH_2_PO_4_, pH 7.4) followed by incubation for 1 h with primary antibody in 1% non-fat powdered milk in PBS. Membranes were then washed three times with PBST (PBS plus 0.1% Tween-20) followed by 1 h incubation with goat anti-rabbit horseradish peroxidase-conjugated secondary antibody (BioRad) in 3% non-fat powdered milk in PBS. The membrane was then washed twice with PBST followed by one wash in PBS before being developed using a Pierce ECL immunoblotting substrate (Thermo Scientific, Stockholm, Sweden) and imaged using a ChemiDoc MP system (BioRad).

## Figures and Tables

**Table 1:** List of oligonucleotides used.

Name	Sequence	Description
Duet_XanL_TEV_Forward	5′-TGAGAATCTTTATTTTCAGGGCAA GCCGGGTAGCCCGAAGAAACGTGGCA	Forward primer to add TEV site to pETDuet-1HvXanL constructs
Duet_XanL_TEV_Reverse	5′-CCCTGAAAATAAAGATTCTCAAG CTTGTCGACCTGCAGGCGCGCCGAGCT	Reverse primer to add TEV site to pETDuet-1HvXanL constructs
DuetTEV2x His_Forward	5′-CACCACCATCATAGCCAGGATCC GAATTCGAGCTCGGCGCGCCTGCAGGT	Forward primer to add 2 extra Histidine residues to make a 8x His tag
DuetTEV2x His_Reverse	5′-GGATCCTGGCTATGATGGTGGTG ATGATGGTGATGGCTGCTGCCCATGGT	Reverse primer to add 2 extra Histidine residues to make a 8x His tag
DuetTEV4x His_Reverse	5′-GGATCCTGGCTATGATGGTGGTGATG ATGGTGATGATGATGGCTGCTGCCCATGGT	Reverse primer to add 2 extra Histidine residues to make a 10x His Tag. This primer was run with DuetTEV2xHis_Forward
